# Revision Surgical Management of Refractory Nasal Vestibular Stenosis in an Exotic Shorthair Cat Using a Combined Surgical Technique and a Steroid-Eluting Implant (PROPEL Contour)

**DOI:** 10.3390/vetsci13050423

**Published:** 2026-04-27

**Authors:** Hyeong-mok Kim, Su-jin Son, Seok-ho Jeon, Hwi-yool Kim

**Affiliations:** 1 Laboratory of Veterinary Surgery, College of Veterinary Medicine, Konkuk University, 120 Neungdong-ro, Gwangjin-gu, Seoul 05029, Republic of Korea; 2 EUM Animal Medical Center, 15 Dongtan-daero 6-gil, Hwaseong-si 18501, Gyeonggi-do, Republic of Korea

**Keywords:** BOAS, Exotic Shorthair, feline, nasal vestibular stenosis, PROPEL Contour, ventral vertical resection

## Abstract

Severe nasal vestibular stenosis in brachycephalic cats involves species-specific anatomical structures distinct from those in dogs, often leading to a high recurrence rate even after standard surgical correction. This case report introduces an innovative surgical strategy for a refractory Exotic Shorthair cat presenting with obligate open-mouth breathing and complete nasal obstruction despite three previous surgical interventions. To remove the recurrent scar tissue and maximize the nasal vestibular lumen, a combined surgical approach was performed, incorporating a ventral vertical resection, bilateral wedge resections, and bilateral single pedicle advancement flaps. To maintain the acquired airway and prevent restenosis, a steroid-eluting bioabsorbable implant (PROPEL Contour) was placed within the resected nasal vestibule, marking its first application in veterinary medicine. Postoperatively, open-mouth breathing resolved immediately. At the long-term 20-month follow-up, normal nasal breathing was maintained without restenosis or nasal discharge, with complete resolution of all preoperative clinical signs, and no implant-related adverse effects or infections were observed. The concurrent application of radical structural resection and a PROPEL Contour implant effectively suppresses tissue regrowth and maintains stable nasal patency, offering a highly promising therapeutic protocol for refractory feline nasal vestibular stenosis.

## Simple Summary

Flat-faced (brachycephalic) cats, such as Exotic Shorthairs, often suffer from severe breathing difficulties due to narrowed nostrils. Because cats have a unique nasal anatomy compared to dogs, standard surgical treatments often fail, leading to a high rate of recurrence as the tissue grows back and blocks the airway again. This report describes an Exotic Shorthair cat that still could not breathe through its nose even after three previous surgeries. To solve this, a novel combination of surgical techniques was used to maximize the opening of the nostrils. Furthermore, to keep the airway open during the healing process, a special dissolving stent (PROPEL Contour) that slowly releases steroids to reduce swelling and scarring was placed inside the nose. This is the first time this implant, typically used in human medicine, has been used in veterinary medicine. The cat’s breathing problems were resolved immediately after surgery. Twenty months later, the cat was breathing normally through its nose with no signs of the blockage returning. This new approach offers a highly effective and permanent solution for flat-faced cats suffering from severe and recurring nasal narrowing, greatly improving their quality of life without the need for multiple surgeries.

## 1. Introduction

Brachycephalic cat breeds, such as the Exotic Shorthair and Persian, have gained immense popularity. Their skulls are characterized by a rounded shape resulting from the extreme shortening of the facial bones and neurocranium, exhibiting infant-like anatomical features. However, these profound morphological alterations driven by selective breeding are a major cause of the increased prevalence of conformation-related disorders. Clinically, this is deeply associated with the development of Brachycephalic Obstructive Airway Syndrome (BOAS) and chronic epiphora, which severely compromise the patient’s quality of life [1].

While BOAS is well-recognized in cats, clinical reports of feline BOAS are less frequent, and the related literature remains very limited. Although an elongated soft palate has been documented in a Persian cat presenting with recurrent dyspnea and pulmonary edema [2], brachycephalic cats generally present primarily with stenotic nares as an isolated clinical sign, lacking the typical multi-level BOAS components seen in dogs.

Notably, the anatomical etiology of stenotic nares differs visually and structurally between cats and dogs. In dogs, stenosis is primarily driven by the axial deviation of the alar wings and alar folds. In contrast, axial deviation is relatively less significant in cats; instead, obstruction is predominantly caused by redundant skin folds at the junction where the ventral floor of the nostril meets the haired skin of the lip, leading to ventral nasal occlusion. To address this feline-specific anatomical difference, surgical approaches such as bilateral single pedicle advancement flaps following the excision of the redundant ventral nasal fold have been attempted to alleviate clinical signs of airway obstruction [3]. Furthermore, radical alaplasty [4] techniques, such as extensive alar tissue resection [5,6], can be considered to maximize the surgical opening. However, relying solely on external tissue excision often fails to prevent restenosis in the complex feline anatomy. Therefore, while a targeted ventral vertical resection can address the specific site of feline obstruction, it inherently requires complementary reconstructive and stenting techniques to maintain long-term patency.

To effectively manage refractory recurrent nasal vestibular stenosis, a PROPEL Contour implant (Medtronic, Minneapolis, MN, USA) was utilized in an off-label capacity in this case. Originally designed to maintain the patency of sinus ostia following functional endoscopic sinus surgery in humans, this bioabsorbable stent is composed of poly(L-lactide-co-glycolide). It acts as a physical scaffold to prevent postoperative adhesions, while simultaneously releasing coated mometasone furoate over approximately 30 days to profoundly suppress local edema and inflammation. Human clinical studies have demonstrated that such drug-eluting stents significantly reduce postoperative scar tissue formation and the need for subsequent surgical interventions [7].

Severe nasal vestibular stenosis and nasal obstruction in brachycephalic cats have a notoriously high recurrence rate following conventional surgical correction due to these species-specific anatomical complexities. The objective of this report is to describe a novel surgical treatment strategy—combining a radical multi-step alaplasty with the insertion of a steroid-eluting implant (PROPEL Contour)—and its clinical outcome in a refractory Exotic Shorthair patient experiencing complete bilateral obstruction at the level of the nasal vestibule despite three previous corrective surgeries.

## 2. Materials and Methods

### 2.1. Case and Ethical Considerations

The medical records of a brachycephalic cat that underwent surgical correction for refractory nasal vestibular stenosis at EUM Animal Medical Center were reviewed. This study involved a non-experimental animal. Established, internationally recognized best practices for veterinary clinical care were followed. Informed verbal consent was obtained from the owner for the procedures performed.

### 2.2. Diagnosis of Refractory Nasal Vestibular Stenosis

Diagnosis was based on the macroscopic evaluation revealing complete bilateral occlusion of the nasal cavities, clinical signs indicative of severe upper airway obstruction (obligate open-mouth breathing and severe dyspnea), and the primary complaint of recurrent stenosis. The patient had a history of three previous failed surgical attempts performed by a referring veterinarian, culminating in total re-occlusion.

### 2.3. Preoperative Evaluation

**Figure 1:**
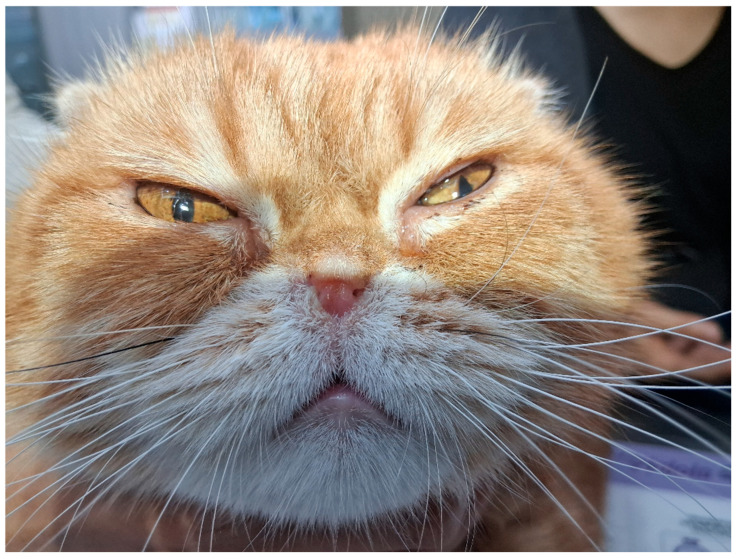
Preoperative appearance demonstrating severe bilateral nasal vestibular stenosis.

Collected data included signalment, clinical history, primary complaints, physical examination findings, and preoperative imaging. The patient was a 4-year-old neutered male Exotic Shorthair weighing 3.3 kg. A significant weight loss (from 4.1 kg to 3.3 kg) was documented over the 6 months prior to presentation. Computed tomography (CT) was utilized to assess structural abnormalities. Imaging revealed severe anterior nasal obstruction characterized by profound soft tissue proliferation extending from the nasal vestibule to the rostral nasal cavity, accompanied by turbinate hypertrophy on the CT images. Macroscopic examination confirmed complete bilateral occlusion of the external nares (Figure 1).

### 2.4. Surgical Technique

The cat was oxygenated for 5 min prior to anesthetic induction. The premedication protocol included midazolam (0.1 mg/kg, IV) (Midazolam Inj.; Bukwang Pharmaceutical Co., Ltd., Seoul, Republic of Korea) and butorphanol (0.2 mg/kg, IV) (Myungmoon Butorphanol tartrate Inj.; Myungmoon Pharm Co., Ltd., Seoul, Republic of Korea). Anesthesia was induced with propofol (3 mg/kg, IV to effect) (Provive; Myungmoon Pharm Co., Ltd., Republic of Korea). During induction, a laryngeal examination was performed to evaluate for the presence of an elongated soft palate and other laryngeal abnormalities. Following the examination, the patient was intubated using a 3.0 mm internal diameter armored endotracheal tube. To minimize airway mucosal irritation, a plain sterile lubricating gel (without lidocaine) was applied to the tube’s cuff prior to insertion. Anesthesia was then maintained with an inhalant anesthetic. The patient was positioned in sternal recumbency with the head slightly elevated using surgical towels placed under the mandible. The head was meticulously positioned to ensure the symmetry of facial features.

Ventral Vertical Resection

**Figure 2:**
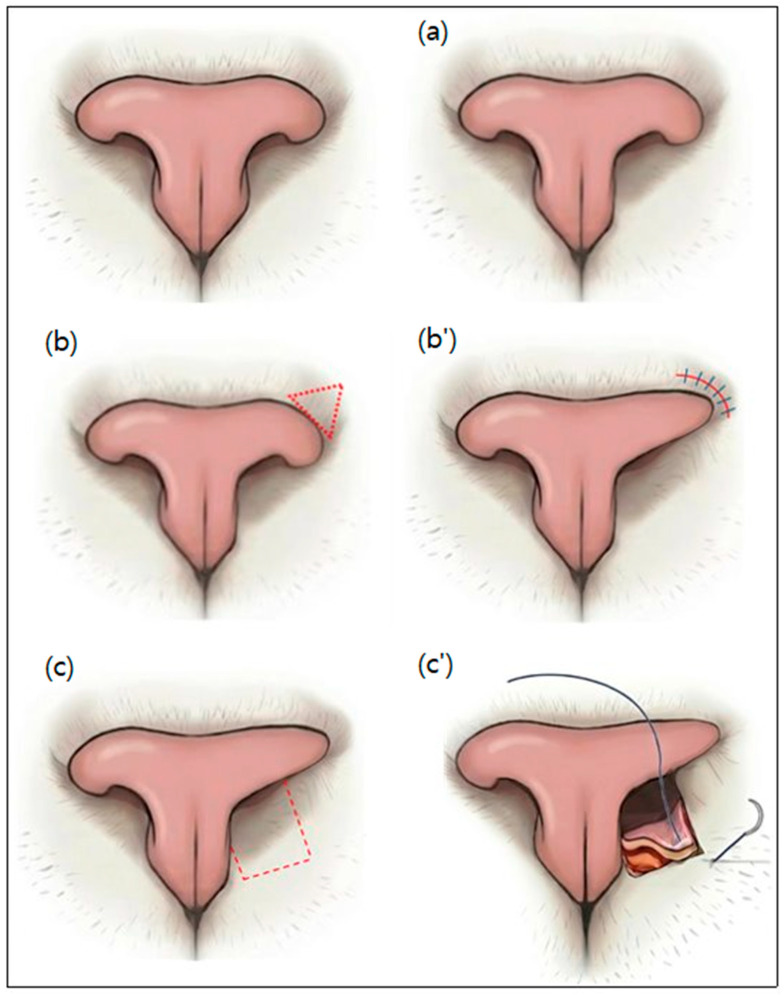
Step-by-step schematic illustrations demonstrating the sequential progression of the combined surgical techniques. The unlabelled top left image represents the preoperative state; (**a**) Ventral vertical resection (5 and 7 o’clock directions); (**b**,**b’**) Subsequent bilateral wedge resections providing strong lateral traction; (**c**,**c’**) Final single pedicle advancement flaps, building upon the state of (**b’**), drawing tissue rostrally to comprehensively maximize the vestibular lumen. The red dashed lines indicate the planned incision margins.

To directly address the feline-specific ventral occlusion, a targeted ventral vertical resection was performed. A No. 11 scalpel blade was directed nearly vertically downward, angled towards the 5 o’clock position for the left nostril and the 7 o’clock position for the right nostril. Single incisions were made at these predetermined angles, effectively resecting the redundant soft tissue and skin folds on the ventral floor of the nares, which are the primary culprits of nasal obstruction in cats (Figure 2a). Hemorrhage was controlled by applying firm, direct pressure to the surgical site for 5 min using sterile cotton swabs soaked in chilled epinephrine solution (1:1000; Bosmin, Jeil Pharmaceutical Co., Ltd., Seoul, Republic of Korea). To maximize the immediate external opening, no sutures were placed at the incision sites, allowing the open wounds to heal naturally by secondary intention through granulation tissue formation and epithelialization.

Bilateral Wedge Resection

Using a No. 11 blade, a full-thickness wedge-shaped excision of the skin and underlying dorsolateral nasal cartilage was performed adjacent to the hairless margins of the nasal planum. Following the wedge resection, the cut margins were approximated and closed with simple interrupted sutures using 5-0 Prolene (Ethicon, Somerville, NJ, USA) (Figure 2b,b’).

Single Pedicle Advancement Flap

Bilateral single pedicle advancement flaps were created using a No. 11 blade. The long axis of the flaps was oriented rostrocaudally. The base of the flap was located just inside the nasal cavity and extended rostrally toward the haired skin of the lip. The width of the flap encompassed the entire ventral floor of the nostril. Redundant ventral nasal folds at the entrance of the nostrils were excised en bloc. The flaps were advanced and closed with simple interrupted sutures using 5-0 Prolene (Ethicon, Somerville, NJ, USA) (Figure 2c,c’).

Placement of a Steroid-Eluting Bioabsorbable Nasal Stent (PROPEL)

**Figure 3:**
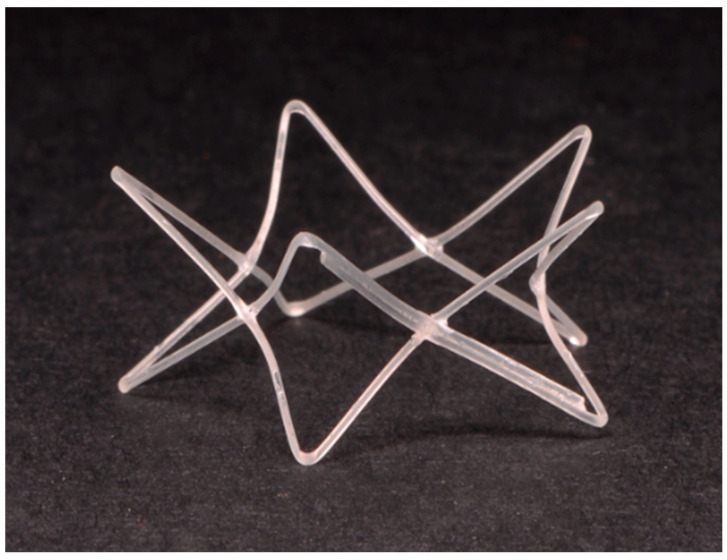
Macroscopic appearance of the PROPEL Contour implant (Dimensions: 8 mm in length and 15.5 mm in diameter). The device is a mometasone furoate-eluting bioabsorbable scaffold with an hourglass configuration.

During this definitive revision surgery, following the physical expansion of the nares achieved by the combined surgical techniques described above (ventral vertical resection, bilateral wedge resections, and pedicle advancement flaps), the internal lumen was sequentially dilated using 4 Fr, 5 Fr, and 6 Fr feeding tubes. The PROPEL Contour implant was then carefully deployed bilaterally intranasally into the resected nares and the medial aspect of the nasal vestibule, extending approximately 8 mm caudally, using its dedicated single-use delivery applicator (Figure 3).

### 2.5. Postoperative Care and Follow-Up

Postoperatively, the patient was admitted to the intensive care unit (ICU) and received 40% oxygen therapy as a routine prophylactic measure against potential post-anesthetic airway edema, and fluid therapy (Hartmann’s solution, 2 mL/kg/hr) for 3 days. Analgesia was provided using meloxicam (0.1 mg/kg, SC, q24h) (Metacam; Boehringer Ingelheim Vetmedica GmbH, Ingelheim am Rhein, Germany) and butorphanol (0.2 mg/kg, IV, q8h). The patient was discharged on postoperative day 3. Throughout the follow-up period, the cat exhibited no signs of foreign body reaction to the implant, such as pawing at the nose, sneezing, or abnormal nasal discharge. Weekly follow-ups were conducted during the first month post-discharge, and a CT scan was performed 3 months postoperatively to evaluate the structural outcome.

## 3. Results

### 3.1. Medical History and Chronological Timeline

The patient had a history of three previous surgical corrections for open-mouth breathing caused by nasal vestibular stenosis, all of which were performed by a referring veterinarian. According to the referral records, the first surgery involved a horizontal wedge resection. Following this initial procedure, the patient remained asymptomatic for approximately 13 months. However, clinical signs recurred, necessitating a second surgery. Due to severe stenosis extending into the internal nasal cavity, a pedicle advancement flap was performed during the second procedure. Unfortunately, one week after the second procedure, open-mouth breathing resumed due to restenosis. A third surgery was performed one month later, utilizing electrocautery to excise the proliferated tissue within the internal nasal cavity to secure patency. Despite this, complete restenosis occurred within 2 weeks. Subsequently, the dyspnea worsened, accompanied by marked anorexia and exercise intolerance, leading to the patient’s referral to our center for a definitive, single-session revision surgery.

### 3.2. Intraoperative Findings and Immediate Postoperative Course

**Figure 4:**
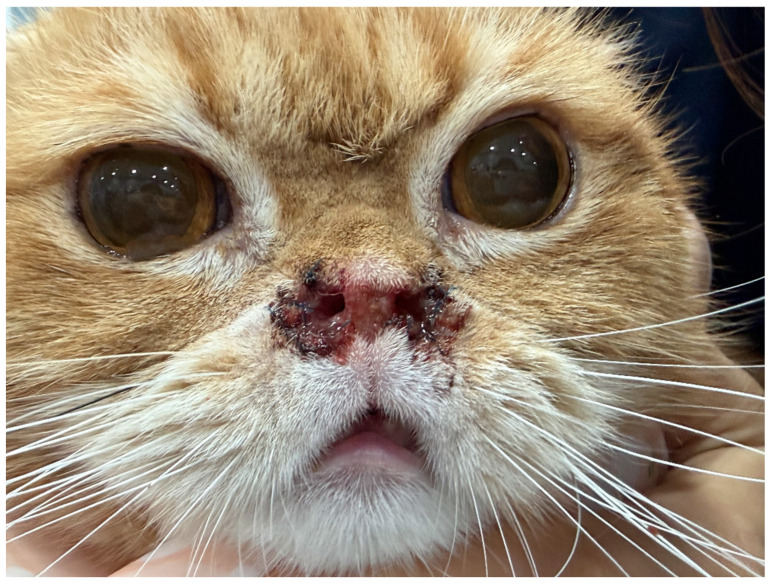
Immediate postoperative view showing the enlarged nares and significantly improved bilateral nasal patency.

A laryngeal examination performed prior to intubation revealed no evidence of an elongated soft palate, laryngeal paralysis, laryngeal collapse, or enlarged tonsils. Immediately following this definitive, single-session combined surgical procedure (multi-step nares expansion concurrently followed by implant placement), the patient’s open-mouth breathing resolved completely (Figure 4).

### 3.3. Postoperative Follow-Up and Prognosis

At 2 weeks postoperatively, the patient was sedated with dexmedetomidine in the preparation room for suture removal, with pre- and post-sedation oxygen supplementation. Laryngeal examination was repeated, revealing no abnormalities such as laryngeal edema or collapse. Following suture removal, a 6 Fr feeding tube was smoothly passed to physically confirm the maintenance of nasal patency. Sedation was successfully reversed with intramuscular atipamezole.

**Figure 5:**
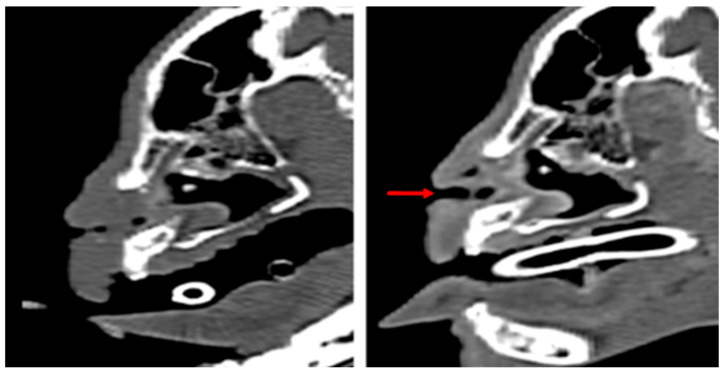
Sagittal CT images comparing the preoperative (**left**) and 3 months postoperative (**right**) airway, showing the resolution of anterior nasal obstruction. The red arrow indicates the maintained patency of the nasal vestibule.

**Figure 6:**
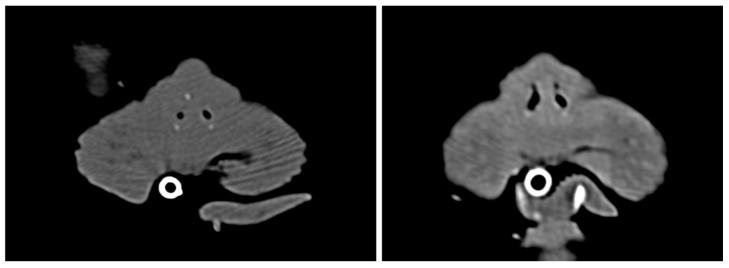
Transverse CT images comparing the preoperative (**left**) and 3 months postoperative (**right**) states, demonstrating maintained luminal patency of the nasal vestibule without tissue regrowth.

At the 2-month recheck, the patient’s body weight had significantly increased from 3.3 kg on the day of surgery to 3.7 kg. The preoperative anorexia and exercise intolerance showed marked improvement starting the day after discharge. A CT scan performed 3 months postoperatively confirmed that the nasal cavity remained normally patent and structurally stable, with no signs of restenosis (Figure 5 and Figure 6).

**Figure 7:**
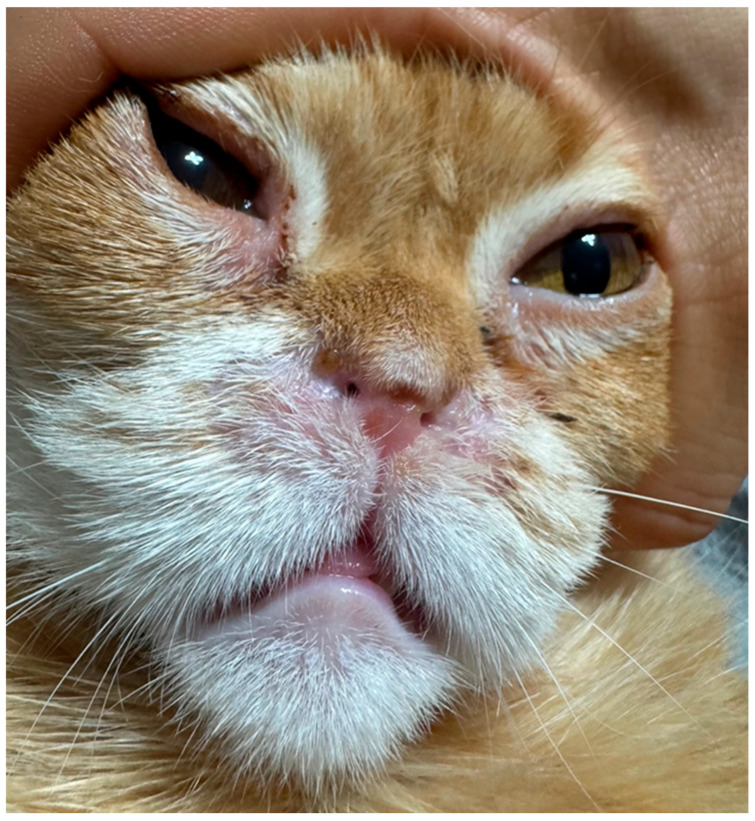
Frontal view of the patient at the 20-month postoperative follow-up. Although noticeable tissue regrowth and a reduction in the external naris diameter are visible at the ventral resection sites compared to the immediate postoperative state (Figure 4), the lateral expansion from the wedge resections is well-preserved. Most importantly, all preoperative clinical signs have completely resolved, indicating maintained internal vestibular patency.

At the long-term follow-up conducted 20 months postoperatively, the owner reported that all preoperative clinical signs, including obligate open-mouth breathing and exercise intolerance, had completely resolved. A recent clinical photograph confirmed the sustained wide patency of the bilateral nasal vestibules without any evidence of functional restenosis. A clinical comparison between the immediate postoperative appearance (Figure 4) and the 20-month follow-up (Figure 7) revealed a reduction in the external naris diameter due to partial tissue regrowth at the ventral resection sites; however, the lateral expansion was maintained, and the patient exhibited unhindered normal nasal breathing. This durable clinical outcome successfully extends well beyond the 13-month recurrence timeline observed after the patient’s initial surgery, confirming the long-term efficacy of this novel combined surgical protocol.

## 4. Discussion

This case represents the first report in veterinary medicine detailing the successful treatment of severe, refractory nasal vestibular stenosis in an Exotic Shorthair cat using a combination of a targeted ventral vertical resection for luminal expansion and the application of a steroid-eluting bioabsorbable implant (PROPEL Contour) to maintain the acquired airway.

As the popularity of brachycephalic feline breeds rises, the clinical significance of feline BOAS is becoming increasingly apparent [8]. Due to extreme morphologic changes in the skull, the facial bones of brachycephalic cats are severely foreshortened, altering the geometry of the nasal airway and causing significant respiratory compromise [9]. The resulting increase in upper airway resistance not only causes dyspnea but can also precipitate fatal secondary complications, such as recurrent pulmonary edema triggered by an elongated soft palate in Persian cats. Therefore, securing unobstructed airflow at the nasal vestibule—the primary gateway of the upper airway—is paramount [2].

However, the pathophysiology of feline nasal vestibular stenosis differs markedly from that of dogs. In dogs, axial deviation of the alar folds is the primary cause of stenosis. In cats, airflow obstruction arises from species-specific, complex etiologies, including redundant ventral skin folds [3] and elongated dorsolateral nasal cartilages [10]. Consequently, applying conventional canine surgical techniques—such as simple wedge resection or ala-vestibuloplasty—to severe feline cases often fails to provide sufficient airflow and results in a refractory course with high recurrence rates due to the complex local anatomy.

To overcome the limitations of conventional techniques, the concept of radical alar amputation, originally described by Trader [5] and validated in Shih Tzus [6], was modified for the feline anatomy to address the vertical occlusion. A synergistic multi-step surgical protocol was applied: ventral vertical resection, bilateral wedge resections, and pedicle advancement flaps. Interestingly, a clinical comparison between the immediate postoperative appearance (Figure 4) and the 20-month follow-up (Figure 7) reveals a visible reduction in the external naris diameter. Specifically, the areas subjected to the ventral vertical resection, which healed by secondary intention, exhibited significant tissue regrowth and scar contracture. This clinical finding highlights the inherent limitation of relying solely on simple excisional techniques for feline nasal vestibular stenosis; while they provide massive initial expansion, they are highly susceptible to potent contractile forces. Conversely, the lateral traction provided by the bilateral wedge resections was relatively well-maintained.

Crucially, despite this partial external restenosis at the ventral aspect, the patient achieved complete and sustained resolution of clinical respiratory signs. This durable clinical success, coupled with the maintained internal spatial expansion observed on the 3-month postoperative CT scans (Figure 5 and Figure 6), indicates that the true core drivers of long-term patency in this multi-modal approach were the pedicle advancement flaps and the PROPEL Contour implant. The single pedicle advancement flaps provided healthy, non-contracting epithelial tissue to functionally reconstruct the ventral defect, acting as a crucial physical barrier against total stricture.

Furthermore, such aggressive surgical resection to maximize the vestibule inevitably leaves extensive exposed wounds. The subsequent healing process—characterized by severe inflammatory responses, excessive granulation tissue formation, and scar contracture—carries an extremely high risk of secondary restenosis. Traditionally, silicone tubes or catheters have been utilized as stents to prevent luminal collapse. Yet, these can incite foreign body reactions, increase secretions, promote mucosal adhesions, and critically require a second general anesthesia for removal [11].

To fundamentally block these complications and stably maintain the maximized vestibular lumen, we implanted the PROPEL Contour into the nasal vestibule. In human otorhinolaryngology, PROPEL is a state-of-the-art device used to maintain airway patency following chronic sinusitis or choanal atresia surgeries. Notably, Galletti et al. reported the successful off-label use of PROPEL in a 7-day-old neonate’s narrow airway to suppress postoperative inflammation and secure physical patency [7].

Introducing PROPEL into the feline nasal vestibule—which is extremely narrow and vulnerable to inflammatory edema, much like a human neonate’s airway—marks the pioneering application of a bioabsorbable steroid stent in veterinary medicine for the management of refractory nasal vestibular stenosis. In this case, PROPEL delivered two powerful synergistic effects. First, its biocompatible, self-expanding mesh structure acted as a mechanical scaffold, flawlessly preventing the physical collapse of the surgically maximized space during the healing phase. Second, as the implant degraded slowly in vivo over approximately 30 days, it continuously released mometasone furoate, a potent topical corticosteroid. This profoundly suppressed acute inflammation and granulation tissue hyperplasia, promoting stable mucosal re-epithelialization. Furthermore, because the implant is naturally bioabsorbed, the need for a secondary anesthetic event for stent removal was completely eliminated, serving as a groundbreaking advantage in reducing patient stress and anesthetic burden [12,13]. Ultimately, it was this stented internal expansion combined with the stable pedicle flaps that successfully neutralized the superficial contracting forces.

It is important to note that the use of the PROPEL Contour implant in felines is off-label. While mometasone furoate acts locally, potential systemic absorption and classic corticosteroid side effects (e.g., polyuria, polydipsia, vomiting, or skin irritation) must be considered. In this patient, however, no systemic or local adverse effects were observed. The gradual, localized release likely minimized systemic bioavailability. Further large-scale studies are warranted to establish safety guidelines for this off-label application in veterinary medicine.

## 5. Conclusions

In conclusion, for refractory nasal vestibular stenosis in brachycephalic cats, an innovative multi-modal protocol was applied. As observed over a 20-month period, natural scar contracture and tissue regrowth following simple excision can partially compromise the initial external surgical expansion. Therefore, we conclude that while initial radical excision (ventral vertical resection and lateral wedge resections) is necessary for maximal immediate opening, the integration of pedicle advancement flaps and an intranasal steroid-eluting stent (PROPEL Contour) is the true critical cornerstone for counteracting internal contracture and sustaining long-term airway patency. This case provides crucial clinical evidence that shifting the surgical focus from mere external excision to ‘flap reconstruction combined with internal stenting’ may serve as a highly promising therapeutic option in feline brachycephalic respiratory surgery.

## Data Availability

The original contributions presented in this study are included in the article. Further inquiries can be directed to the corresponding author.
